# Case Report: Maternal near-miss—recovery from refractory septic shock with multiple organ dysfunction secondary to acute pyelonephritis in pregnancy

**DOI:** 10.3389/fmed.2025.1671969

**Published:** 2025-12-10

**Authors:** Hong Chen, Ziyu Huang, Guihua Chen, Yaling Tang, Dongxia Yang

**Affiliations:** 1 Graduate School, The Second Affiliated Hospital of Heilongjiang University of Chinese Medicine, Harbin, Heilongjiang, China; 2 Department of Obstetrics and Gynecology, The First Affiliated Hospital of Xiamen University, Xiamen, Fujian, China; 3 Department of Clinical Medicine, School of Medicine, Xiamen University, Xiamen, Fujian, China; 4 Department of Obstetrics and Gynecology, Zhangzhou Second Hospital of Traditional Chinese Medicine, Zhangzhou, Fujian, China; 5 Department of Gynecology, The Second Affiliated Hospital of Heilongjiang University of Chinese Medicine, Harbin, China

**Keywords:** urinary tract infections, pregnancy, refractory septic shock, cardiogenic shock, multiple organ dysfunction syndrome, full recovery

## Abstract

**Background:**

Urinary tract infections during pregnancy represent a significant clinical concern, posing considerable risks to both maternal and fetal health. The physiological changes associated with pregnancy increase the susceptibility to infections, with an estimated 15 to 20% of acute pyelonephritis cases potentially progressing to bacteremia, particularly in the context of urinary stones. The onset of sepsis may lead to septic shock and multiple organ dysfunction, adversely affecting the kidneys, liver, lungs, heart, and central nervous system. Recent research into the pathogenesis of sepsis has provided insight into how inflammation and the activation of immune responses can induce abnormalities in circulatory function, subsequently triggering systemic ischemia and resulting hypoxia.

**Case presentation:**

A 42-year-old pregnant woman presented with acute renal colic and fever, leading to the diagnosis of nephrolithiasis, hydronephrosis, and acute pyelonephritis. The clinical condition rapidly deteriorated, evolving into uroseptic shock, cardiogenic shock, and multiple organ dysfunction syndrome. This alarming progression necessitated her admission to the intensive care unit, where she received aggressive treatment interventions, including extracorporeal membrane oxygenation (ECMO), an intra-aortic balloon pump (IABP), and continuous renal replacement therapy (CRRT). Remarkably, these life-saving measures proved successful. After nine days in intensive care, she fully recovered and was discharged.

**Conclusion:**

This case study explores three critical clinical challenges: strategies for early identification of high-risk pregnant women for sepsis; optimal timing for interventions in obstructive urinary tract infections to prevent septic shock and multi-organ dysfunction; and the implementation of mechanical circulatory support. The circulatory support protocol highlights the importance of personalized interventions in complex medical scenarios.

## Background

Urinary tract infections (UTIs) during pregnancy constitute a significant clinical concern, presenting risks to both maternal and fetal health. The hormonal alterations associated with pregnancy lead to the relaxation of smooth muscle in the ureters, and the mechanical compression from an enlarging uterus further increases the susceptibility to renal pelvis dilation. This combination of factors substantially heightens the incidence of UTIs in pregnant women (1, 2). Approximately 15 to 20% of pregnant women diagnosed with acute pyelonephritis (APN) may exhibit progression to bacteremia (3). The concomitant presence of urinary stones, particularly in the context of obstructive factors, markedly elevates the risk of sepsis. Research has indicated that this risk can increase up to threefold (4). This condition creates a detrimental cycle characterized by infection, obstruction, and a systemic inflammatory response, thereby exacerbating the complexity and severity of the clinical scenario.

Sepsis is life-threatening organ dysfunction caused by a dysregulated host response to infection, early identification and appropriate management in the initial hours after the development of sepsis improve outcomes (5). The new proposed WHO definition of maternal sepsis is “a life-threatening condition defined as organ dysfunction resulting from infection during pregnancy, childbirth, post-abortion, or postpartum period” (6). In recent decades, significant advancements have been made in understanding of the pathophysiology of sepsis. Researches have clarified the mechanisms by which the activation of the immune response leads to substantial dysfunction in both macro- and microcirculation, ultimately resulting in widespread systemic hypoperfusion. Notably, sepsis has the potential to trigger multiple organ dysfunction, with the kidneys, liver, lungs, heart, central nervous system, and hematological system being the most frequently affected. Clinically, the state of systemic hypoperfusion may present through characteristic signs, including hypotension, prolonged capillary refill time, mottled skin, and cool extremities (7, 8). The cornerstone of multiple organ dysfunction resulting from sepsis is the sustained imbalance between perfusion and the metabolic demands of tissues. In the pathological process, the dysfunction of the cardiac system, incited by inflammatory mediators, alongside the redistribution of systemic blood volume, plays a critical role. Moreover, the diminished capacity of tissues to utilize oxygen intensifies the existing predicament. This mechanism is primarily driven by inflammation-mediated cardiac dysfunction and the abnormal distribution of systemic blood volume, further worsened by impaired utilization of oxygen by tissues. Multiple organ dysfunction syndrome (MODS), a predominant manifestation of sepsis, frequently serves as a critical determinant of patient outcomes (7, 9, 10).

This case report delineates the successful multidisciplinary management of a distinctive obstetric patient who, during her second trimester, developed nephrolithiasis and acute pyelonephritis, culminating in a cascade of life-threatening complications including septicemia, refractory septic shock, cardiogenic shock, and multiple organ dysfunction syndrome. This case is particularly notable for its unique constellation of factors: the convergence of a high-risk pregnancy, obstructive uropathy, and the rapid progression to a triple shock state (septic, cardiogenic, and distributive) with MODS. Through this singular case, we aim to elucidate three pivotal clinical challenges, each highlighted by the specific particularities of this presentation: (1) the imperative for early recognition of sepsis in pregnant women with specific risk constellations, such as advanced maternal age and obstructive calculi; (2) the determination of the critical intervention window for obstructive UTIs to avert the irreversible progression to combined shock and MODS; and (3) the strategic implementation and sequencing of advanced mechanical circulatory support (VA-ECMO, IABP, and CRRT) in a peripartum context, amidst the concomitant challenges of obstetric hemorrhage and fetal demise. The stepwise hemodynamic support protocol employed herein, which was instrumental in achieving complete maternal recovery following this catastrophic clinical course, offers a critical framework for managing similarly complex scenarios. It underscores the necessity of a tailored, dynamic, and multidisciplinary approach in critical care obstetrics.

## Case presentation

**Figure 1:**
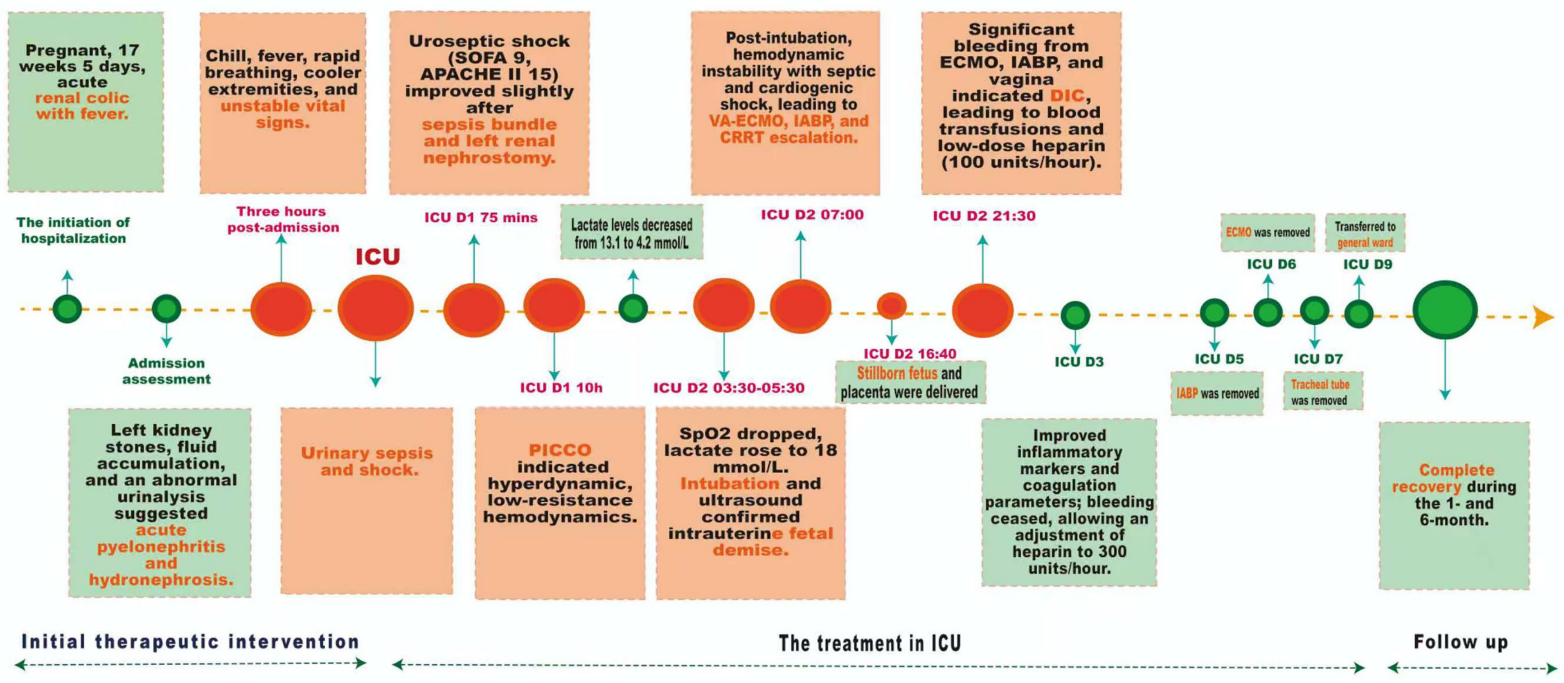
The timeline of treatment.

A 42-year-old female with a height of 163 cm and a weight of 67.6 kg, was admitted with left renal colic and fever during 17 weeks and 5 days of pregnancy. Vital signs were stable, with a temperature of 36.4 °C, pulse of 86 bpm, blood pressure of 125/78 mmHg, and respiratory rate of 20 bpm. Tenderness was noted in the left renal region, and the abdomen was soft and non-distended. The fetal heart rate was elevated at 178 bpm. Ultrasound confirms an intrauterine pregnancy and multiple calculi in the left kidney’s renal pelvis, with the largest measuring 1.5 cm×0.9 cm. There was also localized fluid accumulation in the left renal pelvis, suggestive of hydronephrosis or parapelvic cysts. Laboratory investigations showed leukocytosis and elevated inflammatory markers (The white blood cell count was 13.98 × 10^9/L, the percentage of neutrophils had increased to 87.6%, and the C-reactive protein level was 43.2 mg/L). Urinalysis revealed significant abnormalities: red blood cells at 134.2/uL, white blood cells at 1324.3/uL, bacterial counts at 76,420.9/uL, urine ketones, and 3 + occult blood. Liver and kidney function tests were normal. Preliminary diagnosis included multiple stones in the left kidney, acute pyelonephritis, and hydronephrosis. The patient was administered second-generation cephalosporin antibiotics (intravenous cefuroxime, 1.5 grams, twice daily) along with antispasmodics; however, there was no significant improvement in symptoms observed. As shown in Figure 1 of treatment, three hours post-admission, the patient developed chills, fever (38.9 °C), shortness of breath, and cooler extremities. Vital signs were unstable: heart rate 116–179 bpm, blood pressure 96–125/61–80 mmHg, respiratory rate 22–28 breaths per minute, with oxygen saturation at 97% on high-flow nasal oxygen. Urinary sepsis and shock were suspected; fluid resuscitation, escalated antibiotics, and vasopressors were administered before transferring the patient to the intensive care unit (ICU) for advanced management.

The patient in the ICU was conscious but restless, with tachypnea, a temperature of 39.4 °C, blood pressure at 70/40 mmHg, and a heart rate of 170–180 bpm (Figure 1). Despite high-flow oxygen, oxygen saturation was 100%. The patient had a Sequential Organ Failure Assessment (SOFA) score of 9, an Acute Physiology, Chronic Health Evaluation (APACHE) II score of 15, an obstetrically modified quick SOFA (omqSOFA) score of 3, and an obstetrically modified SOFA (omSOFA) score of 7, indicating uroseptic shock. The sepsis bundle intervention included deep venous access, norepinephrine, β-lactam antibiotics, and vancomycin, along with aggressive fluid resuscitation. An ultrasound-guided left renal nephrostomy drained light red urine, modestly improving the condition. On the first day in the ICU, the patient’s initial urine output was low, ranging from 30 to 100 mL per hour. After 10 h in the ICU, the patient remains hypotensive at 88/54 mmHg despite norepinephrine infusion at 0.5 μg/kg/min. Pulse indicator continuous cardiac output (PICCO) monitoring shows a hyperdynamic, low-resistance hemodynamic state. Following intervention, lactate levels decreased from 13.1 mmol/L to 4.2 mmol/L. In the ICU D2 (03:30–05:30), the patient experienced a sudden drop in blood oxygen to 83% and a lactate level of 14.5 mmol/L. After adjusting non-invasive positive pressure ventilation, oxygen levels improved to 90%, but lactate rose to 18.0 mmol/L, indicating worsening condition. Intubation was decided, and anti-infection treatment was continued. An ultrasound re-examination revealed intrauterine fetal demise. 1.5 h post-intubation, the patient displayed significant hemodynamic instability, despite receiving high-dose norepinephrine at a rate of 2.0 μg/kg/min. Laboratory analyses revealed elevated lactate levels, while echocardiographic evaluation indicated impaired systolic function, with blood pressure readings fluctuating between 80–95/40–45 mmHg. The clinical findings suggested a complex condition indicative of both septic and cardiogenic shock, coupled with multi-organ dysfunction. In response to this critical state, the management strategy was promptly escalated to include veno-arterial extracorporeal membrane oxygenation (ECMO), an intra-aortic balloon pump (IABP), and continuous renal replacement therapy (CRRT). A stillborn fetus and placenta were delivered, with clear, odorless amniotic fluid and a blood loss of 400 mL in the afternoon. Ultrasound showed no significant residual tissue. That evening’s assessment revealed notable hemorrhaging from the ECMO, IABP catheter, and the vaginal site, raising the suspicion of disseminated intravascular coagulation (DIC). In response, active blood transfusions were commenced, alongside a low-dose heparin infusion administered at a rate of 100 units per hour to facilitate effective anticoagulation management. On the third day of ICU treatment, inflammatory markers declined significantly, with no bleeding and improved coagulation. Considering the pregnant patient on V-A ECMO and IABP therapy, anticoagulation was adjusted to low-dose heparin at 300 units per hour. After active treatment, the patient’s condition improved significantly. By day five in the ICU, the IABP was removed, hemodynamic management was optimized, blood pressure stabilized, and sedation was reduced. On day six, infection was controlled, allowing for ECMO removal. By day seven, tubes were removed, sedation ceased, and a follow-up cranial CT scan showed normal results. The patient was discharged to the ward on day 9 and demonstrated a complete recovery during the 1- and 6-month follow-up.

## Discussion

APN during pregnancy is a serious urinary tract infection with significant incidence and mortality rates, leading to frequent prenatal hospitalizations (1). The pathophysiological mechanisms of APN are closely tied to anatomical changes and hormonal shifts occurring during pregnancy (1, 2). These changes elevate the risk of urinary tract infections and systemic inflammation. Nephrolithiasis during pregnancy increases the risk of acute pyelonephritis due to kidney stones obstructing urine flow, leading to retention and epithelial damage that favor bacterial adherence and biofilm formation (4). The patient developed kidney stones causing hydronephrosis and urinary obstruction, leading to urosepsis, septic shock, cardiogenic shock, and MODS, resulting in intrauterine fetal demise. This case highlights the risks of acute pyelonephritis during pregnancy, especially with urinary tract stones, and the challenges of managing sepsis in critical situations. Pregnancy-related infections, a leading direct cause of maternal mortality, are responsible for a significant number of deaths defined as those from any pregnancy-related cause during or within 42 days of termination of pregnancy. Sepsis associated with APN is a major contributor to this global burden (11, 12). Clinical evidence has suggested that approximately 15 to 20% of individuals diagnosed with APN may progress to sepsis (3). This systemic infectious response poses substantial risks to both maternal and fetal wellbeing through a myriad of mechanisms: microcirculatory disruptions, endothelial damage, and hemodynamic instability can precipitate septic shock, DIC, and acute respiratory distress syndrome (ARDS). Furthermore, inadequate placental perfusion directly contributes to adverse pregnancy outcomes, including preterm birth, fetal growth restriction, and intrauterine fetal demise (13, 14). In this instance, the patient was diagnosed with infections caused by Staphylococcus aureus and Escherichia coli, as identified through pathogen-specific blood cultures and urine cultures collected via nephrostomy. Furthermore, subsequent blood metagenomics next-generation sequencing (mNGS) testing offered additional confirmation of the implicated pathogens. Research has shown that lipopolysaccharide, an important component of the outer membrane of Gram-negative bacteria, is one of the key triggers of septic shock in ICU patients (15). Further research has found that changes in Gram-negative bacteria in the bloodstream can trigger a series of systemic inflammatory responses, which often lead to severe or even fatal consequences (16). Despite the patient receiving standardized and aggressive anti-infection and anti-shock treatment, the condition still progressed to refractory septic shock. Even with sufficient doses of vasopressor agents (norepinephrine doses exceeding 0.5 μg/kg/min), the patient continued to experience tissue hypoperfusion and end-organ dysfunction (e.g., sustained hypotension, elevated lactate levels, and organ failure). A key feature of refractory shock is severe tissue perfusion inadequacy, along with significant cellular and metabolic failure. Existing evidence from evidence-based medicine indicates that the prognosis for such patients is significantly dose-dependent on the need for vasopressor agents: when the equivalent dose of norepinephrine exceeds 1 μg/kg/min, the mortality rate can sharply rise to 80–90% (15, 17).

**Figure 2:**
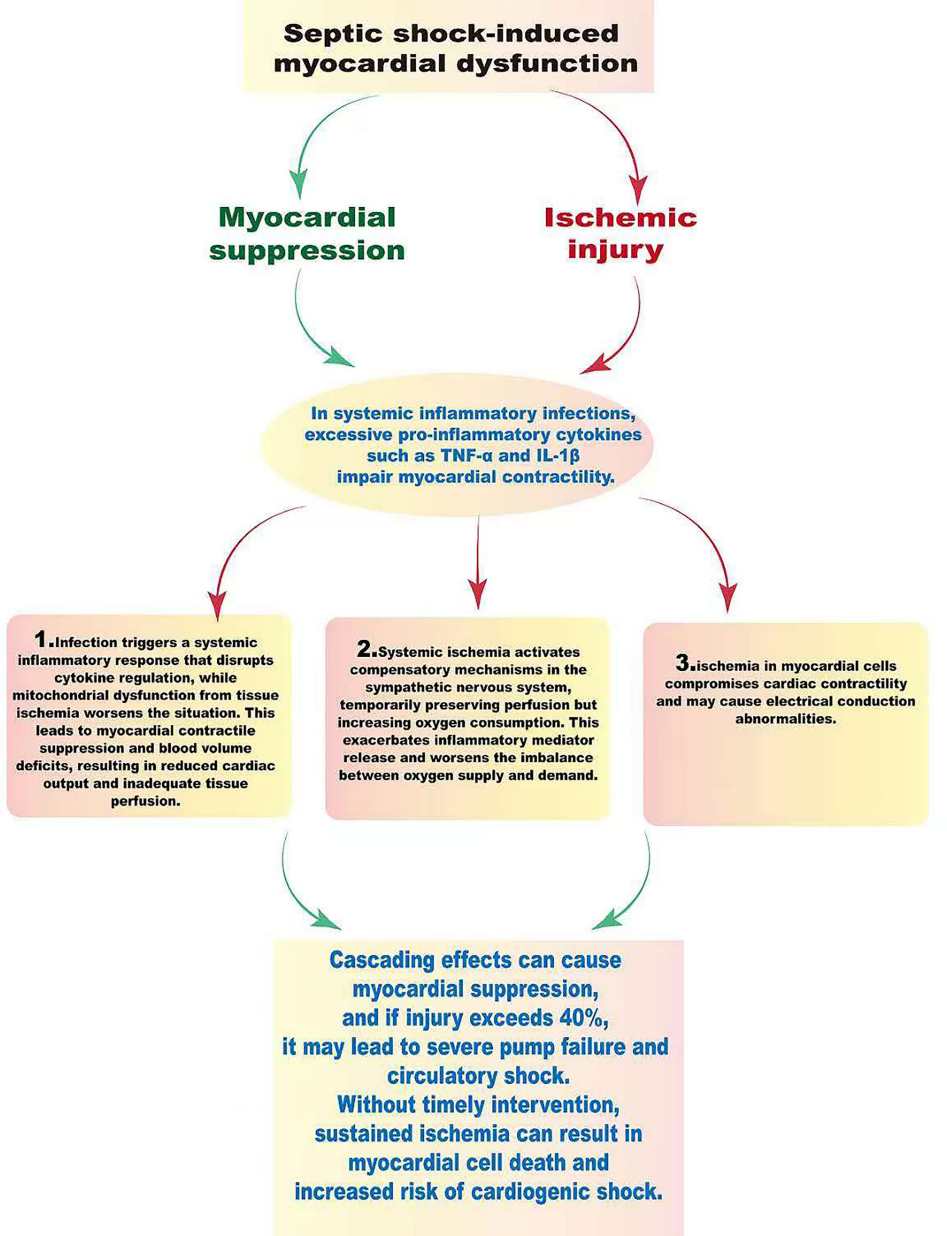
The pathophysiological mechanisms of septic shock and myocardial dysfunction. Septic shock is primarily characterized by circulatory dysfunction due to excessive vasodilation, leading to significant hemodynamic changes and decreased blood pressure. This inflammatory response, triggered by infection, results in the release of cytokines like TNF-α and IL-1β, worsening myocardial suppression and ischemia. Cardiac dysfunction progresses through three stages: the first involves decreased myocardial contractility and reduced blood volume, leading to lower cardiac output and impaired tissue perfusion. In the second stage, systemic ischemia activates the sympathetic nervous system, which temporarily maintains perfusion but increases oxygen consumption and inflammatory mediator release, worsening the oxygen supply–demand imbalance. The third stage features ischemic damage to myocardial cells, decreased contractility, and electrical conduction disturbances, possibly culminating in cardiac pump failure and circulatory collapse. Prolonged ischemia can lead to irreversible cell death and myocardial infarction, complicating the state of cardiogenic shock.

According to the updated definition for 2024, the essence of sepsis resides in the dysregulation of the host response, which manifests as pathological damage across multiple dimensions, including immune-mediated resistance, disease tolerance, tissue repair capacity, and the mechanisms of inflammation resolution (18). Researches have underscored that the critical importance of maintaining a dynamic equilibrium between pro-inflammatory and anti-inflammatory responses throughout the anti-infection processes; this balance is vital not only for the effective clearance of pathogens but also for the immune system’s restoration of homeostasis. However, patients suffering from sepsis often present with pronounced immune dysregulation. Specifically, excessive inflammatory responses may incite a cytokine storm, consequently leading to microvascular thrombosis and potentially escalating into septic shock and multiple organ dysfunction. Conversely, the immune resistance process is characterized by a multitude of energy-intensive biosynthetic pathways that are essential for the generation of the molecular defenses necessary for host protection, thereby imposing a significant metabolic burden (19–21). The fundamental pathophysiological mechanism underlying septic shock is characterized by circulatory dysfunction. An exaggerated inflammatory response triggers the release of nitric oxide (NO), which induces prolonged relaxation of vascular smooth muscle, culminating in a state of vasodilatory shock. Hemodynamic assessments may reveal a paradoxical presentation of elevated cardiac output coupled with a diminished systemic vascular resistance index (SVRI) (5, 22, 23). As shown in Figure 2, septic shock-induced myocardial dysfunction is a complex process marked by myocardial suppression and ischemic injury due to inflammatory agents. In infections triggering a systemic inflammatory response, excessive pro-inflammatory cytokines like tumor necrosis factor (TNF)-α and interleukin (IL)-1β directly compromise myocardial contractility, clinically evident as a reduced left ventricular ejection fraction (LVEF) (7, 24). This complex process involves three key pathological phases. Initially, an infection triggers a systemic inflammatory response that dysregulates cytokine release, while mitochondrial dysfunction from tissue ischemia worsens the condition. These effects lead to myocardial contractile suppression, absolute and relative blood volume deficits, resulting in decreased cardiac output and poor tissue perfusion. In the second phase, systemic ischemia sparks compensatory mechanisms in the sympathetic nervous system, which might temporarily sustain perfusion but increase oxygen consumption, perpetuating the release of inflammatory mediators and worsening the imbalance between oxygen supply and demand. Finally, ischemia in myocardial cells compromises cardiac contractility and may cause electrical conduction abnormalities. This cascading effect can lead to severe myocardial suppression and, if the injury exceeds 40%, results in severe pump failure and circulatory shock. Without intervention, persistent ischemia may cause myocardial cell death and irreversible necrosis, known as myocardial infarction, significantly contributing to cardiogenic shock (25). VA-ECMO is indicated for refractory circulatory failure and severe cardiogenic shock (LVEF < 25% or cardiac index < 1.5 L/min/m^2^) in septic shock patients (26, 27). In this case, VA-ECMO was initiated due to poor left ventricular function and inadequate response to high-dose vasoactive therapy. It provides full cardiac output, improving oxygen delivery as indicated by SvO₂. Careful anticoagulation adjustment is essential in patients with DIC to manage bleeding risks. The IABP served as a crucial adjunct, enhancing coronary perfusion during diastole and reducing left ventricular afterload in systole, which decreases myocardial oxygen consumption. The combination of VA-ECMO and IABP can effectively lower left ventricular afterload and aid in myocardial recovery, supported by evidence-based medicine. A retrospective study has revealed that among patients with sepsis and cardiogenic shock, the 30-day survival rate was higher in those receiving both ECMO and IABP (52%) compared to ECMO alone (33%) (27, 28). This highlights the value of early combined VA-ECMO and IABP therapy in disrupting the cycle of inflammation, myocardial suppression, and circulatory collapse, offering critical rescue for severely ill patients. The implementation of this comprehensive treatment strategy should be based on precise hemodynamic assessment and adjusted according to the individual circumstances of the patient.

**Figure 3:**
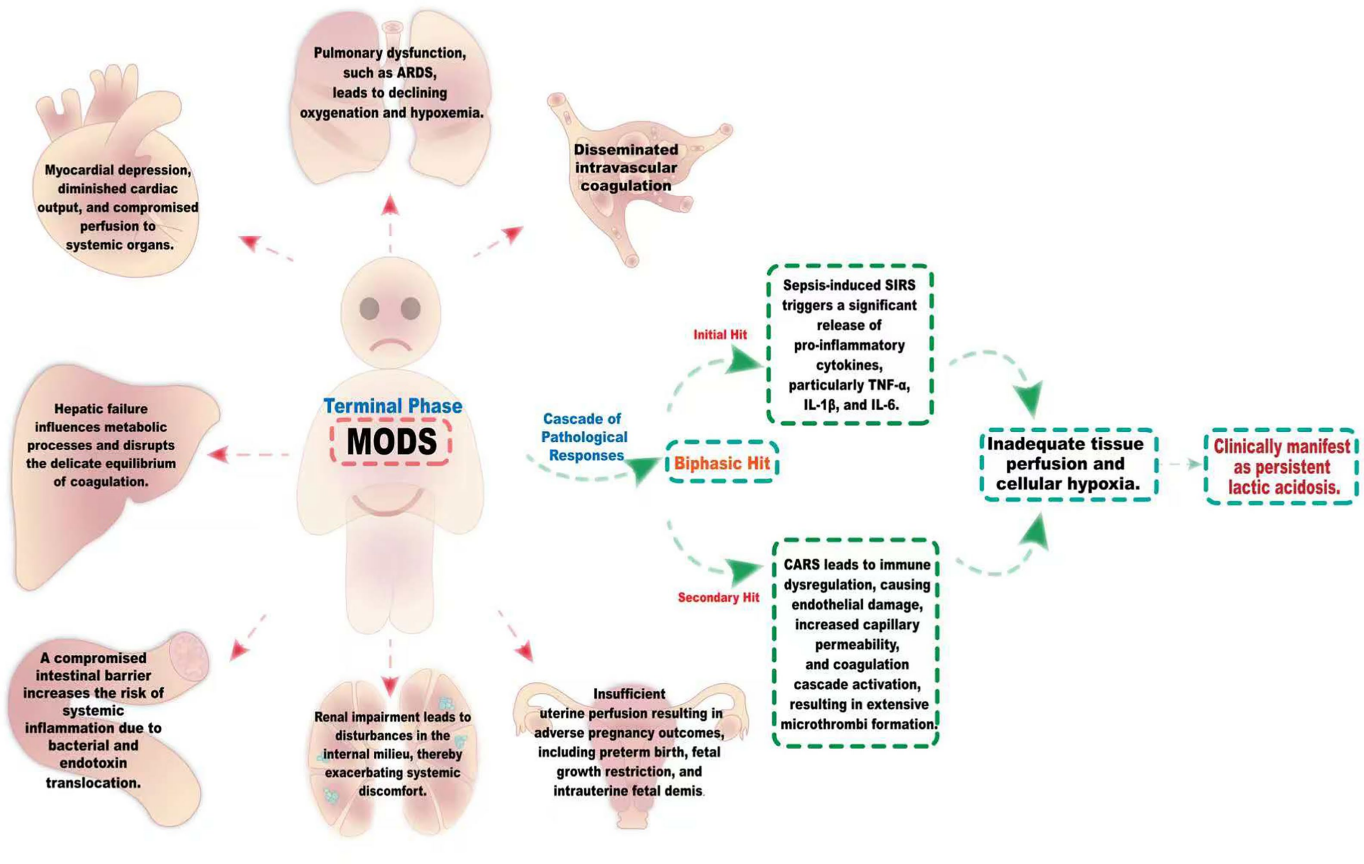
The intricate pathological network of MODS induced by sepsis. The pathogenesis of this syndrome follows a biphasic hit model. During the initial hit phase, the systemic inflammatory response syndrome (SIRS) induced by sepsis triggers a marked release of pro-inflammatory cytokines, notably TNF-α, IL-1β, and IL-6. Subsequently, in the secondary hit phase, a state of immune dysregulation emerges, resulting from the compensatory anti-inflammatory response syndrome (CARS). Sepsis-induced MODS encompasses a sophisticated interplay among the lungs, heart, liver, kidneys, and gastrointestinal systems. The onset of ARDS results in inadequate oxygenation, leading to hypoxemia, which in turn impairs myocardial performance and diminishes cardiac output. This cascade of events compromises organ perfusion. Concurrently, liver dysfunction disrupts both metabolic and coagulation homeostasis, markedly elevating the risk of hemorrhage. Meanwhile, renal insufficiency induces imbalances in the internal milieu, intensifying overall discomfort. The deterioration of intestinal barrier integrity heightens the translocation of bacteria and endotoxins, culminating in a pronounced systemic inflammatory response. These pathological alterations synergistically exacerbate the patient’s condition, thereby significantly complicating therapeutic interventions.

MODS represents a critical manifestation in the terminal phase of sepsis, characterized by a complex cascade of pathological responses. As shown in Figure 3, the pathogenesis of this syndrome adheres to a biphasic hit model. In the initial hit phase, the sepsis-induced systemic inflammatory response syndrome (SIRS) triggers a significant release of pro-inflammatory cytokines, particularly TNF-α, IL-1β, and IL-6. Subsequently, during the secondary hit phase, a state of immune dysregulation arises, attributable to the compensatory anti-inflammatory response syndrome (CARS). At the microcirculatory level, endothelial cell damage leads to capillary leakiness and the activation of the coagulation cascade, culminating in the extensive formation of microthrombi. Collectively, these processes contribute to inadequate tissue perfusion and cellular hypoxia, which clinically manifest as persistent lactic acidosis (29, 30). The intricate interplay among various organ systems manifests as a sophisticated pathological network, characterized by a mechanism of inter-dialogue. Pulmonary dysfunction, exemplified by conditions such as ARDS, often precipitates progressive hypoxemia, which in turn induces myocardial depression, diminished cardiac output, and compromised perfusion to systemic organs. Furthermore, hepatic failure exerts profound effects on metabolic processes and the delicate balance of coagulation. Renal impairment leads to disturbances in the internal milieu, thereby exacerbating systemic discomfort. The compromise of the intestinal barrier not only undermines its defensive capacity but also facilitates the translocation of bacteria and endotoxins, establishing a damaging cycle. The synergistic promotion of these pathological alterations markedly heightens the complexity of therapeutic intervention (7, 30–33).

In this case, the tragic outcome of fetal demise is closely tied to the mother’s severe progression of septicemia, which was precipitated by a significant reduction in placental perfusion and subsequent severe fetal hypoxia due to maternal circulatory failure. This situation underscores the dangers associated with septicemia during pregnancy and emphasizes the need for early intervention. Initially diagnosed with acute pyelonephritis complicated by obstruction upon admission, the patient’s early treatment with a second-generation cephalosporin proved insufficient in managing her condition. This highlights the necessity for broader and more robust initial empirical antibiotic therapy for pregnant patients presenting with high-risk factors such as obstruction, with subsequent modifications based on antibiotic sensitivity testing, as illustrated by the later identified mixed infection. The evolution of the mother’s condition serves as a cautionary example, swiftly transitioning from a localized infection to the critical triad of septic shock, cardiogenic shock, and multiple organ dysfunction syndrome. A pivotal moment in her treatment was the implementation of an aggressive, multidisciplinary life support strategy. This approach included addressing the source of infection through nephrostomy, in conjunction with the use of VA-ECMO, IABP, and CRRT, effectively breaking the cycle of inflammation, myocardial suppression, and circulatory collapse. Furthermore, it is crucial to consider the regulation of immune balance. Early identification of high-risk patients is fundamental to preventing and managing this syndrome, thereby enabling prompt interventions that can disrupt the inflammatory cascade. Concurrently, providing personalized support for the various organ systems is vital to enhancing patient prognosis and improving overall outcomes.

## Conclusion

This clinical example illustrates the formidable challenge posed by physiological changes during pregnancy, which often predispose patients to urinary tract infections that can swiftly escalate to septic shock. The individual in this case not only contended with multiple renal stones but also developed acute pyelonephritis, culminating in the rapid onset of septic shock, cardiogenic shock, and multiple organ dysfunction—a perilous “triple hit.” Through the concerted efforts of a multidisciplinary team, a robust response was mounted employing a phased hemodynamic support strategy: the initial phase prioritized the optimization of vasopressor agents, while the subsequent phase introduced mechanical circulatory support. Furthermore, meticulous attention to the source of infection was instrumental in transcending the constraints of conventional bundled treatment approaches, thereby facilitating the implementation of a holistic strategy encompassing “mechanical support enhancement + targeted infection management + pregnancy-specific care,” which effectively ameliorated the patient’s condition. In conclusion, this case emphasizes the critical importance of recognizing the unique vulnerabilities associated with urinary tract infections when confronting sepsis during pregnancy. Future investigations should aim to establish an obstetric-specific early warning system, delve into the intricacies of immune regulatory mechanisms during pregnancy and the differential responses to vasopressor agents, and pursue phenotype-targeted therapies alongside biomarker-guided individualized strategies to enhance treatment outcomes for high-risk populations.

## Data Availability

The original contributions presented in the study are included in the article/supplementary material, further inquiries can be directed to the corresponding authors.
